# Does Childhood Adversity Lead to Drug Addiction in Adulthood? A Study of Serial Mediators Based on Resilience and Depression

**DOI:** 10.3389/fpsyt.2022.871459

**Published:** 2022-04-18

**Authors:** Jingzhen He, Xinyu Yan, Rufang Wang, Juyou Zhao, Jun Liu, Changwei Zhou, Yumei Zeng

**Affiliations:** ^1^Health Psychology Institution, Chengdu University of Traditional Chinese Medicine, Chengdu, China; ^2^Institute of Brain and Psychological Sciences, Sichuan Normal University, Chengdu, China; ^3^Rehabilitation Department, Sichuan Drug Rehabilitation Administration, Chengdu, China; ^4^Psychological Correction Center, Sichuan Ziyang Drug Rehabilitation Center, Ziyang, China

**Keywords:** adverse childhood experiences, drug addiction, resilience, depression, mediating effect

## Abstract

Drug addiction is a common problem worldwide. Research has shown adverse childhood experiences (ACEs) to be an important factor related to drug addiction. However, there are few studies on how ACEs lead to drug addiction and the role of resilience and depression in this process. Thus, the main purposes of the study were to determine the proportion of those with adverse childhood experiences who take drugs in adulthood and how resilience and depression affect this relationship. The results showed that (1) greater severity of ACEs made individuals more likely to take drugs; (2) ACEs were positively correlated with depression, and resilience was negatively correlated with ACEs and depression; and (3) ACEs not only affected drug addiction through resilience or depression alone but also through the combined action of resilience and depression, indicating that depression led to drug addiction while resilience weakened the effect of ACEs on depression and drug addiction. Furthermore, in the serial mediation model, abuse, neglect, and family dysfunction were significant predictors of drug addiction. Our results are encouraging in that they provide guidance in understanding the complex relationships among ACEs, resilience, depression, and drug addiction.

## Introduction

Adverse childhood experiences (ACEs) are typically defined as stressful and/or traumatic experiences that occur during childhood (1, 2). A study have shown that more than 60% of adults report having at least one adverse childhood experience, and 17% report four or more adverse childhood experiences (3). There is increasing evidence that adults with ACEs are at greater risk for diseases (e.g., alcoholism, myocardial infarction, stroke, depression, diabetes, and coronary heart disease) and disability due to health status (4–8). Moreover, ACEs are a major risk factor for drug abuse. For instance, childhood abuse is closely related to marijuana use (9, 10). Individuals with ACE scores ≥5 are seven to 10 times more likely to report illicit drug addiction compared to those without ACEs (11), and are four to 12 times more likely to become drug abusers (6). In short, ACEs not only affect physical and mental health but also increase the risk of drug abuse in adulthood.

Depression is one of the most common and main negative emotions induced by ACEs. Compared with other negative emotions, the impact of depression on drug addiction has more important clinical significance. Many studies have identified a relationship between ACEs and depression, as adults with ACEs are more likely to suffer from depression compared to adults without such experiences (12–15). Emotional, sexual, and physical child abuse are the most important risks factors for depression (12). A retrospective cohort study showed that the risk of depressive disorders increased for decades after ACEs (16). Compared with adults without ACEs or those who have not experienced trauma in adulthood, individuals with ACEs (including sexual and physical abuse) are more likely to suffer from long-term PTSD and depression; simultaneously, they are more likely to take drugs, use more types of drugs, and have more serious drug dependence (17, 18). Thus, there is a noticeable relationship between ACEs and depression. Further, multiple studies have uncovered the comorbidity of depression and drug addiction; that is, depression can lead to drug addiction, and drug addiction can lead to or exacerbate depression (19–21). Drug-addicted individuals tend to express themselves negatively, and negative stimulation can aggravate their negative emotions and exacerbate drug abuse (22, 23). Avoidance of negative affect is the predominant motive for drug abuse (24).

Resilience is a dynamic process in which individuals can adaptively overcome stress and/or traumatic events (25). It is the ability to overcome life challenges with perseverance, self-awareness, and one's own internal coherence by activating a personal growth project (26). ACEs may produce negative outcomes, such as depression; however, some individuals with ACEs will bounce back rather than suffer long-term negative consequences, and they are considered to have better resilience (27). It is beneficial to help individuals establish and improve resilience and to promote mental health education interventions, which facilitate recovery from trauma and stress and mitigate the influence of ACEs on depression (28, 29).

**Figure 1:**
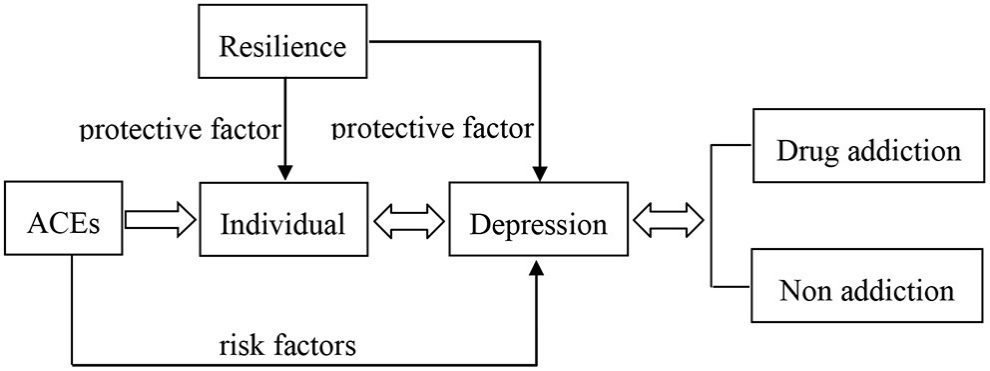
The relationship between ACEs, drug addition, depression, and resilience.

In summary, there is a strong relationship between ACEs and drug addiction. ACEs can produce and exacerbate depression, and depression may be an important cause of drug abuse. Additionally, resilience seems to impact the relationship between ACEs, depression and drug addiction. However, how ACEs affect drug addiction directly is much less studied, and the roles of resilience and depression in drug addiction are still unclear. Therefore, this study first examined the relationship between ACEs and drug addiction and then examined resilience and depression as potential contributors of this relationship. In order to show the complicated relationship between ACEs, drug addition, depression and resilience more clearly, a graphic illustration is created in Figure 1.

## Methods

### Participants

We used random sampling to recruit 937 participants including 459 individuals with drug addictions (252 males, 207 females) and 478 individuals without them (138 males, 340 females). Those with drug addictions were recruited from two drug rehabilitation centers in Sichuan Province, China. Approximately 70% of this group were methamphetamine addicts and the rest were heroin, Magu, and K powder addicts. Those without drug addiction were also recruited from southwest China^1^.

All participants whom we recruited met the following criteria: (1) age 18–50 years, (2) no serious mental illness, and (3) educational background of elementary school or above. Moreover, participants with drug addictions met the DSM-V diagnostic criteria for psychoactive substance abuse or dependence, completing physiological detoxification and providing negative urine tests. All participants provided informed consent before beginning the study.

## Measures

### Adverse Childhood Experiences

We used the ACEs questionnaire to collect information on participants' exposure to ACEs (prior to age 18). The questionnaire consists of 28 items divided into three categories and 10 subscales, which include childhood abuse (emotional, physical, and sexual), childhood neglect (emotional and physical), and growing family dysfunction (substance abuse, mental illness, domestic violence, criminal household members, and parental marital discord). One ACE was recorded for each subscale that met the conditions of exposure to ACEs. We used the ACE scores (10 ACEs subscales; 0-10 possible ACEs) to evaluate the cumulative effect of multiple ACEs, with higher ACE scores indicating more serious exposure to ACEs (31). ACE scores can be divided into four levels according to the degree of ACE exposure: no exposure = 0 ACEs; mild = 1-2 ACEs; moderate = 3–4 ACEs; and severe ≥5 ACEs. In this study, the Cronbach's alpha value for the ACEs questionnaire was 0.629.

### Connor-Davidson Resilience

We measured the resilience of participants over the past month using the Connor-Davidson Resilience Scale (CD-RISC), which consists of 25 items scored on 5-point Likert scales ranging from 1 (not at all) to 5 (almost exactly). Connor and Davidson proposed the five-factor scoring method to differentiate the five dimensions of resilience (32): F1 (personal ability, high standards, and tenacity), F2 (belief in instincts, tolerance of negative events, and resistance to stress), F3 (active acceptance of change and secure relationships), F4 (control), and F5 (religious influence). Higher scores indicate better resilience, and total scores range from 1 to 105. The scale's Cronbach's alpha was 0.913.

### Depressive Symptoms

The Beck Depression Inventory (BDI) is a self-report questionnaire with 21 items, which we used to assess participants' degree of depression. Each item is rated from 0 to 3, yielding lowest and highest possible total scores of 0 and 63, respectively (33). Higher total scores indicate higher degrees of depression. The scale has demonstrated satisfactory test-retest reliability and internal consistency. To improve the structural equation model's fit and control the multi-item measurement error of latent variables, we used the factor balance method to package the 21 single-dimensional items into three indicators (D1, D2, D3), with each indicator containing seven items (34, 35). The BDI's Cronbach's alpha value was 0.916.

### Procedures

Before starting the survey, we informed all participants that all data collected from them would remain confidential and be used for scientific research purposes only. All who met the inclusion criteria signed informed consent before voluntarily participating in the survey. Participants completed the ACEs questionnaire, CD-RISC, and BDI separately, which took them a total of 25–30 min. We collected and checked the completed questionnaires on site and distributed small gifts as compensation.

### Data Analysis

We performed data preprocessing, χ2-tests, analysis of variance (ANOVA), and correlation analysis in SPSS 23.0 (χ2-tests for categorical variables and ANOVA for continuous variables). Additionally, we conducted structural equation modeling (SEM) analyses in Mplus 8.3. We used the robust weighted least squares estimation (WLSMV) extraction procedure to test the model fit to the data. The WLSMV does not assume normally distributed variables and provides the best option for modeling categorical or ordinal data (36, 37). Further, we used bias-corrected bootstrap analysis with 1,000 bootstrap samples to test the mediating effect.

We utilized an item parceling strategy to control the multi-item inflation error of the latent variables (35). Specifically, we divided the unidimensional BDI into three indicators using the factor balance method. Drug addiction was treated as a dummy variable in the mediation model. As recommended by Hu and Bentler (38), a model is considered to fit the data well if the standardized root mean square residual (SRMR) and the root mean square error of approximation (RMSEA) values are below 0.08 and the comparative fit index (CFI) and Tucker-Lewis index (TLI) values are above 0.90.

## Results

### Sample Description

Table 1 shows the demographic characteristics of participants. ACE exposure levels of participants were as follows: no exposure (ACE score = 0; *n* = 203), mild exposure (ACE score = 1–2; *n* = 396), moderate exposure (ACE score = 3–4; *n* = 215), and severe exposure (ACE score ≥ 5; n = 123). There were no significant differences in the average age of participants across ACE exposure levels (*F* = 0.89, *p* = 0.45). The number of participants with drug addictions who were exposed to severe ACEs was higher than those without exposure (84 vs. 71; *p* < 0.001). Additionally, CD-RISC scores decreased with increased ACE exposure levels (86.12 vs. 85.31 vs. 82.74 vs. 79.65; *p* < 0.01); In contrast, higher ACE exposure levels were associated with higher BDI scores (8.35 vs. 10.95 vs. 15.38 vs. 20.20; *p* < 0.001).

**Table 1:** Characteristics of sample and ACE group comparisons for all variables.

	**Total** ***N* = 937**	**ACE group (*n*)**				**χ^2^, *F*** **(*p*)**
		**No exposure** **(203)**	**Mild exposure** **(396)**	**Moderate exposure** **(215)**	**Severe exposure** **(123)**	
Age (M ± SD)	28.51 ± 11.10	29.43 ± 11.69	28.07 ± 11.14	28.83 ± 11.35	27.82 ± 9.34	*F* = 0.89 (*p* = 0.45)
Sex-Female (%)	547 (58.4)	142 (70.0)	235 (59.3)	113 (52.6)	57 (46.3)	χ^2^ = 21.67 (*p* < 0.001)
Addiction (%)	459 (48.99)	71 (40.6)	172 (47.9)	131 (64.2)	84 (70.2)	χ^2^ = 51.66 (*p* < 0.001)
CD-RISC (M ± SD)	83.75 ± 14.86	86.12 ± 14.84	85.31 ± 14.56	82.74 ± 14.98	79.65 ± 13.95	*F* = 6.59 (*p* < 0.001)
BDI (M ± SD)	12.62 ± 11.07	8.35 ± 8.91	10.95 ± 10.21	15.38 ± 10.81	20.20 ± 12.48	F = 41.49 (*p* < 0.001)

## Correlational Results

### Relationship Between ACE Exposure and Drug Addiction

**Figure 2:**
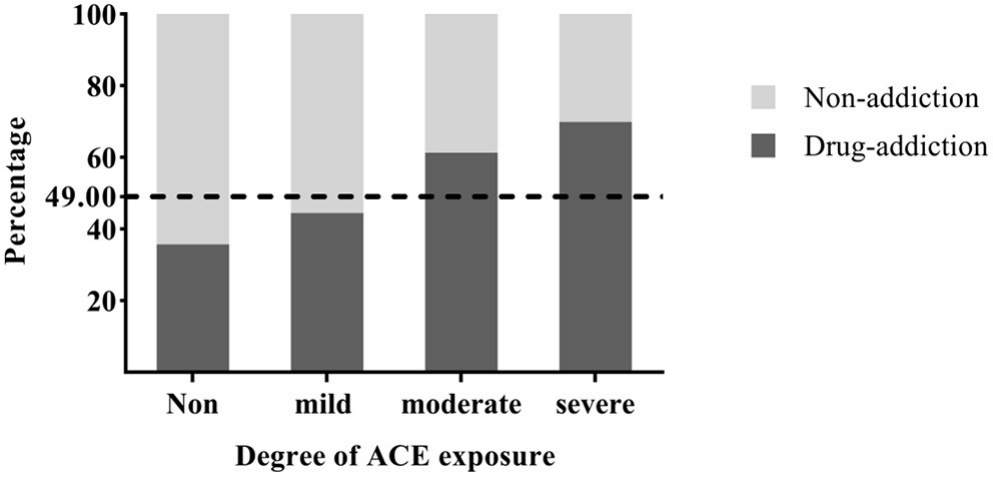
Proportions of drug users with different ACE exposure levels. The dotted line represented the proportion of participants with drug addiction in all.

A bar chart (Figure 2) was used to show the proportion of drug users reporting different ACE exposure levels. We designated the degree of ACE exposure as the abscissa and the rates of drug addiction and non-addiction as the ordinate, as shown in Figure 2. With increased ACE exposure levels, the rate of drug addiction also increased, which indicated that the more serious ACEs participants suffered, the more likely they were to take drugs. Similarly, higher ACE exposure levels were associated with lower rates of non-addiction.

### Correlational Analysis

There were significant correlations among all variables (Table 2). ACEs (childhood abuse, childhood neglect, and family dysfunction) and resilience were negatively correlated. ACEs (childhood abuse, childhood neglect and family dysfunction) and BDI scores were positively correlated. Additionally, resilience was negatively correlated with BDI scores.

**Table 2:** Correlations among ACEs, resilience, and depression.

	**1**	**2**	**3**	**4**	**5**	**6**
1. ACEs	-					
2. Childhood Abuse	0.74^**^	-				
3. Childhood Neglect	0.59^**^	0.27^*^	-			
4. Family Dysfunction	0.83^**^	0.35^**^	0.25^**^	-		
5. Resilience	−0.15^**^	−0.07^*^	−0.21^**^	−0.09^**^	-	
6. BDI	0.35^**^	0.28^**^	0.22^**^	0.27^**^	−0.26^*^	-

* *p < 0.05,*

** *p < 0.01*.

### Confirmatory Factor Analysis

We used confirmatory factor analysis to test whether the measurement model adequately fit the sample data. Two latent variables were included in the full model (resilience and depression) along with eight observed variables. Results showed that the measurement model fit the data well (χ2 (19) = 64.181, CFI = 0.989, TLI = 0.984, RMSEA = 0.050, SRMR = 0.024). All factor loadings were significant (*p* < 0.001), indicating that the structural equation model could be used in the next step of the analysis.

### Common Method Bias Test

The questionnaires used in our survey were self-report, so we also conducted principal components analysis with all questionnaire items (i.e., a common method bias test). Based on the Harman single-factor method, we contend that common method bias was negligible because the variance of the maximum factor interpretation was 16.185, which is less than 40% (39).

### Structural Equation Model

**Figure 3:**
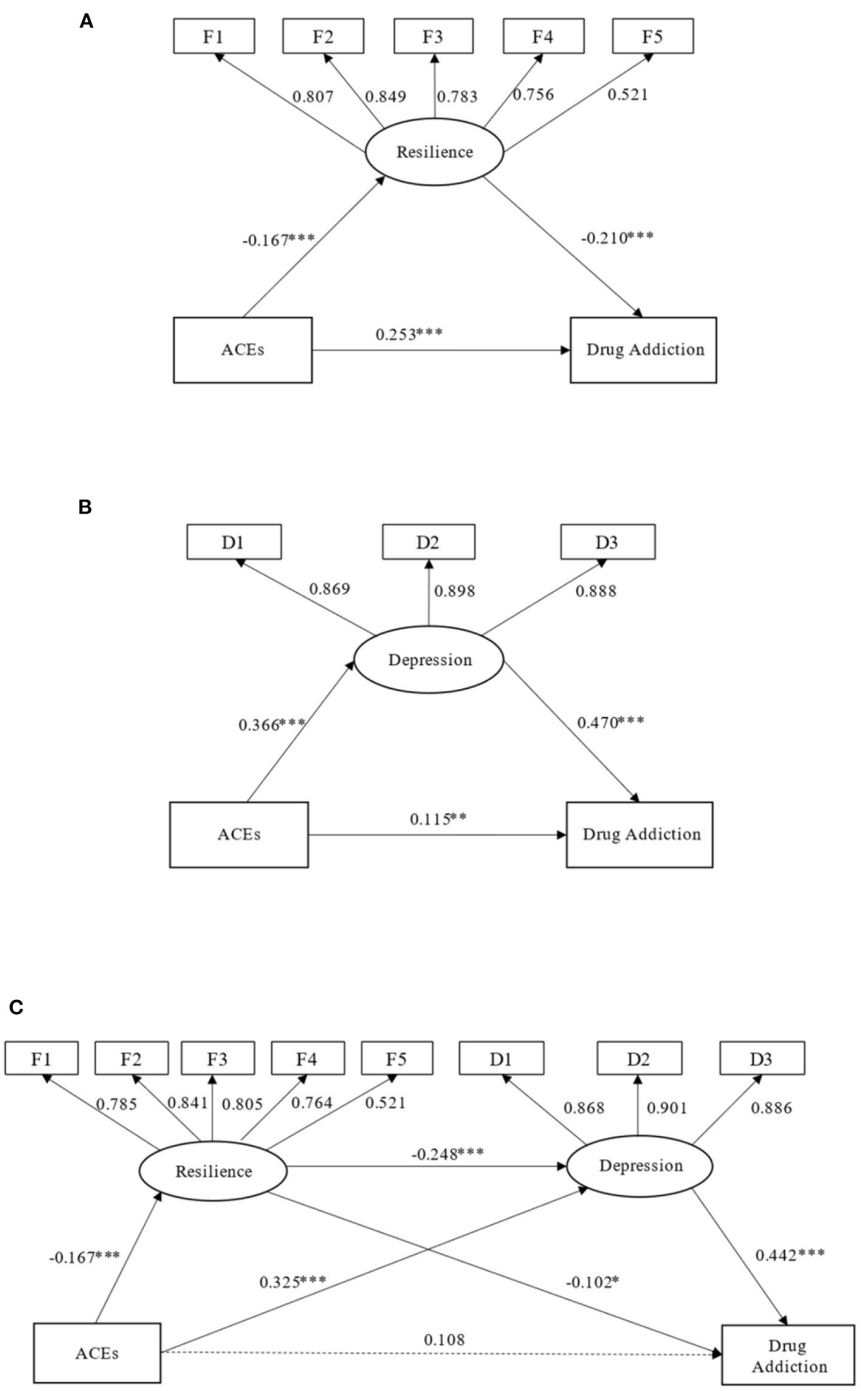
Single-factor mediation models (Models 1 and 2) were established with resilience **(A)** or depression **(B)** as the mediator, respectively. Model 3 was established with resilience and depression as serial mediators **(C)**. Path coefficients are standardized. *p < 0.05, **p < 0.01, ***p < 0.001.

First, we found that the direct effect of the predictor (ACEs) on the dependent variable (drug addiction) in the model without mediators was significant (β = 0.288, *p* < 0.001, 95% CI = 0.207 to 0.366). Next, we built Model 1 and Model 2 with resilience (M1) and depression (M2) as the respective mediators. The bias-corrected bootstrap analyses (1,000 samples) showed that both mediating effects were significant (Figure 3).

Based on the single-factor mediation model results, we established a serial mediation model with resilience and depression as the serial mediators (Figure 3C). This structural equation model fit the data well, χ^2^(31) = 166.199, CFI = 0.938, TLI = 0.909, RMSEA = 0.068, SRMR = 0.039. In the serial mediation model, ACEs were negatively associated with resilience (β = −0.167, *p* < 0.001, 95% CI = −0.234 to −0.098) and positively associated with BDI scores (β = 0.325, *p* < 0.001, 95% CI = 0.252 to 0.388) and drug addiction (β = 0.108, *p* < 0.001, 95% CI = 0.018 to 0.193); resilience was negatively associated with BDI scores (β = −0.248, *p* < 0.001, 95% CI = 0.367 to 0.527) and drug addiction (β = −0.102, *p* = 0.016); and BDI was positively associated with drug addiction (β = 0.442, *p* < 0.001). As presented in Table 3, the indirect effect of resilience and depression as serial mediators in the relationship between ACEs and drug addiction was significant (β = −0.010, *p* < 0.001, 95% CI = 0.005 to 0.016). Moreover, the mediating effects of resilience (β = 0.009, *p* < 0.001, 95% CI = 0.002 to 0.019) and depression (β= 0.078, *p* < 0.001, 95% CI = 0.057 to 0.100) were also significant.

**Table 3:** Indirect effects with bootstrap 95% CIs.

**Model**	**Pathway**	**Estimate**	**Bootstrap 95% CI**	***p***
Model 1	ACEs → Resilience → Drug Addiction	0.019 (0.006)	0.009, 0.032	<0.001
Model 2	ACEs → BDI → Drug Addiction	0.093 (0.012)	0.072, 0.115	<0.001
Model 3	ACEs → Resilience → Drug Addiction	0.009 (0.004)	0.002, 0.019	0.036
	ACEs → BDI → Drug Addiction	0.078 (0.011)	0.057, 0.100	<0.001
	ACEs → Resilience → BDI → Drug Addiction	0.010 (0.003)	0.005, 0.016	0.003
Model 4	Abuse → Resilience → Drug Addiction	0.011 (0.007)	0.002, 0.029	0.101
	Abuse → BDI → Drug Addiction	0.156 (0.024)	0.112, 0.203	<0.001
	Abuse → Resilience → BDI → Drug Addiction	0.014 (0.006)	0.003, 0.027	0.022
Model 5	Neglect → Resilience → Drug Addiction	0.040 (0.017)	0.010, 0.076	0.019
	Neglect → BDI → Drug Addiction	0.137 (0.030)	0.083, 0.197	<0.001
	Neglect → Resilience → BDI → Drug Addiction	0.046 (0.011)	0.028, 0.071	<0.001
Model 6	Family dysfunction → Resilience → Drug Addiction	0.010 (0.005)	0.002, 0.023	0.061
	Family dysfunction → BDI → Drug Addiction	0.098 (0.016)	0.068, 0.131	<0.001
	Family dysfunction → Resilience → BDI → Drug Addiction	0.011 (0.004)	0.003, 0.019	<0.001

**Figure 4:**
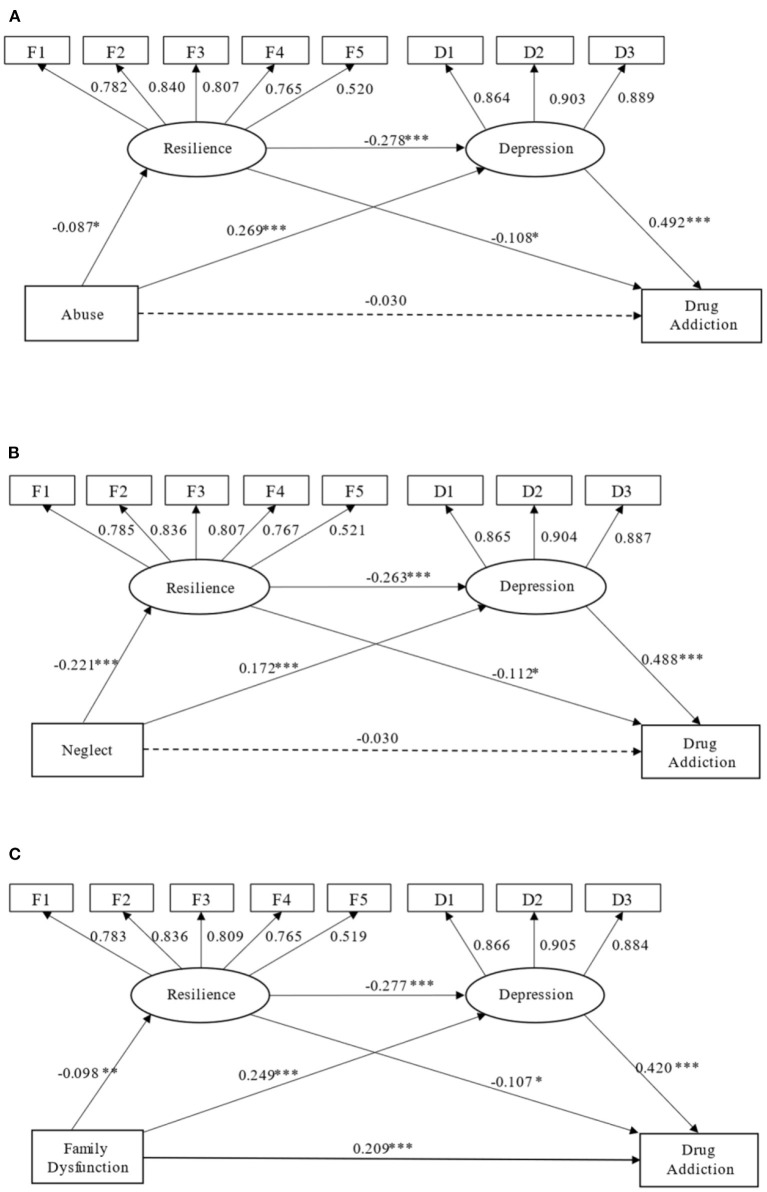
**(A–C)** Serial mediation models (Models 4–6) were established with different subcategories of ACEs (childhood abuse, childhood neglect, family dysfunction) as their respective predictors. Path coefficients are standardized. *p < 0.05, **p < 0.01, ***p < 0.001.

As ACEs included three subcategories (childhood abuse, childhood neglect, and household dysfunction), we built additional serial mediation models accordingly (Models 4–6). Results showed acceptable fit for these three models (CFI = 0.916 to 0.922, TLI = 0.887 to 0.900, RMSEA = 0.056 to 0.068, SRMR = 0.039 to 0.055). Further, the indirect effect of resilience and depression as serial mediators in the relationship between the subcategories of ACEs (childhood abuse, childhood neglect, and family dysfunction) and drug addiction were all significant (β = 0.011 to 0.046, *p* < 0.001). Specifically, the mediating effect of resilience was significant only when childhood neglect was the predictor (*p* = 0.019). Figure 4 provides further information.

## Discussion

Many previous studies have demonstrated the close relationship between ACEs and drug addiction (6, 9–11) and the significant comorbidity of depression and drug addiction (19–21). Our research results also support this. However, how ACEs affect drug addiction and the relationships among ACEs, depression, and drug addiction remain unclear. Therefore, we established a serial mediation model including ACEs, depression, and drug addiction to clarify their relationships (Figure 3). Our research showed that ACEs may not lead directly to drug use but may lead to depression, which in turn leads to drug addiction. Additionally, we showed that resilience played a mediating role between ACEs, depression, and drug addiction (Figure 3C). It showed that improving the resilience levels of people can not only directly mitigate drug use, but also weaken the effect of depression on drug addiction, which provided a guidance for the clinical treatment of drug addicts patients to some extent.

### Drug Addiction Often Associated With More Severe ACE Exposure

As shown in Figure 2, more serious exposure to ACEs yielded higher rates of drug addiction. This is consistent with previous research results (11). In other words, as exposure to ACEs increased, rates for non-addiction decreased significantly, which may explain why some people use drugs to alleviate the negative effects of childhood trauma to some extent. Namely, those who have suffered from severe ACEs might not have been able to address their negative consequences until adulthood (6), choosing to use drugs to reduce the stress or trauma (24).

### The Negative Role of Depression in the Choice on Whether to Use Drugs

The results indicated that the direct effect of ACEs on drug addiction was not significant. However, we found a significant indirect effect in the relationship between ACEs and drug addiction in this study (Figure 3C). ACEs significantly affected depression, which increased the likelihood of drug use. This also supports Farrugia's results showing that individuals with ACEs were more likely to suffer from depression and to use drugs (18). Additionally, the results showed that childhood abuse, childhood neglect, and family dysfunction all significantly affected depression, in turn affecting drug use (Figure 4). Notably, among the three subcategories of ACEs, family dysfunction not only directly affected drug addiction but also indirectly affected drug addiction through depression (Figure 4C), illustrating that the substance abuse, mental illness, domestic violence, criminal household members, and parental marital discord experienced in childhood were more likely to lead to depression in adulthood. For example, parents' drug abuse increases their children's risk for major depression later in life (40). Children are more likely to have ACEs and increased risk for depression if they have alcohol-abusing parents (41). Domestic violence is strongly associated with depression, and it is an indicator of increased exposure to other forms of adversity (14).

### Resilience Mitigates Drug Use

Our study found that resilience played a significant mediating role with respect to ACEs, depression, and drug addiction (Figure 3C). Resilience weakened the effect of ACEs on drug addiction. On the other hand, more serious exposure to ACEs led to lower resilience. Meanwhile, resilience was negatively correlated with depression. Resilience weakened the impact of ACEs on depression and then weakened the effect of depression on drug addiction. The protective role of resilience against depression has been reported previously. For instance, whether in childhood or adulthood, emotional regulation can effectively reduce the negative effects of ACEs and promote physical and mental health (42, 43). Resilience interventions can reduce the impact of ACEs (28). Further, early recognition of ACEs, teaching resilience, and health education can reduce the trauma, stress, and other behavioral and emotional consequences of ACEs (44).

### Limitations and Future Directions

The results of the current study must be interpreted in light of several limitations. First, there are many factors affecting whether an individual takes drugs, and experiencing ACEs may be only one of them. Second, the potential for recall bias is inevitable when participants recall childhood experiences, which may have affected the accuracy of the results. Additionally, self-reports of ACEs are likely to lead to inconsistencies due to underreporting (45). Third, depression may be only one of many negative emotions caused by ACEs, which could make us ignore the impact of other negative outcomes of ACEs on drug addiction. Therefore, future research should explore the impact of multiple factors on drug addiction, the psychosocial mechanism of resilience and how to improve it to combat negative emotions optimally.

## Conclusions

Exposure to ACEs was significantly associated with drug addiction in our study. The more serious ACE exposure was, the more likely it was to lead to drug addiction. ACEs affected drug addiction through depression, and there was a significant correlation between depression and drug addiction. As a protective factor, resilience reduced the effect of ACEs on drug addiction and the effect of depression on drug addiction by reducing the effect of ACEs on depression. Therefore, we should pay more attention to the possible negative effects of ACEs, especially depression. Simultaneously, we should aim to prevent ACEs from the outset. Moreover, we should support ACE sufferers' mental health. Practitioners should provide resilience skills training for those with ACEs to improve their resilience levels and mitigate drug abuse and other negative consequences as much as possible.

## Data Availability Statement

The raw data supporting the conclusions of this article will be made available by the authors, without undue reservation.

## Ethics Statement

All survey processes involving human participants were reviewed and approved by the review committee of Sichuan Drug Rehabilitation Administration. All participants provided informed consent before participation.

## Author Contributions

RW designed research, collected data, and conceptualized the study. JH conceptualized the study, performed literature review, wrote the article, and revised the article. XY analyzed data and revised the article. JZ and JL provided resources and opinions. CZ and YZ organized investigation and data curation. All authors contributed to this manuscript and approved the submitted version.

## Funding

This study was supported by the National Social Science Fund of China (17XSH010).

## Conflict of Interest

The authors declare that the research was conducted in the absence of any commercial or financial relationships that could be construed as a potential conflict of interest.

## Publisher's Note

All claims expressed in this article are solely those of the authors and do not necessarily represent those of their affiliated organizations, or those of the publisher, the editors and the reviewers. Any product that may be evaluated in this article, or claim that may be made by its manufacturer, is not guaranteed or endorsed by the publisher.

## Footnotes

^1^The sample size of this study was determined according to the research on childhood adversity increases the risk of substance (30). According to G^*^power, we need at least 853 sample sizes to maintain power values above 0.9. Therefore, we investigated a total of 937 samples, which was enough to infer the relationship between ACEs and drug addiction, and the power values of *post hoc* test was 0.92.
